# Poly(Vinyl Alcohol)/Hyaluronic Acid Nanofibers for Biomedical Use Under Physiological Conditions: Electrospinning Fabrication and Stabilization via Solvent-Free Citric Acid Crosslinking

**DOI:** 10.3390/polym18010079

**Published:** 2025-12-27

**Authors:** Gianluca Ciarleglio, Nicholas Capuccilli, Elisa Toto, Maria Gabriella Santonicola

**Affiliations:** Department of Chemical Engineering Materials Environment, Sapienza University of Rome, Via del Castro Laurenziano 7, 00161 Rome, Italy; gianluca.ciarleglio@uniroma1.it (G.C.); capuccilli.1797862@studenti.uniroma1.it (N.C.); elisa.toto@uniroma1.it (E.T.)

**Keywords:** nanofibers, electrospinning, poly(vinyl alcohol), hyaluronic acid, citric acid, wound dressings

## Abstract

Electrospun polymeric nanofibers have emerged as promising materials for wound management owing to their high surface area, efficient exudate absorption and gas exchange, and extracellular-matrix-like architecture. This study investigates the fabrication of nanofiber dressings from poly(vinyl alcohol) (PVA) and hyaluronic acid (HA), prepared by fully aqueous electrospinning (without organic solvents) for potential wound-care applications. HA incorporation is expected to influence hydration and matrix interactions, properties that have been associated with modulation of wound healing in previous studies. However, the high solubility of PVA-based NFs in aqueous environments limits their use in biological applications. To address this issue, PVA/HA nanofibers were chemically crosslinked through a solid-state esterification process at 150 °C using biocompatible citric acid (CA). The electrospinning parameters were optimized to obtain PVA/HA fibers with diameters ranging from 130 to 200 nm, which were assembled to form mats with different porosity and intersection density. FTIR confirmed the formation of ester bonds, while DSC analysis showed an increase in Tg from 41 °C to about 55 °C and a slight decrease in Tm after crosslinking. Swelling and degradation analyses demonstrated a significant enhancement in hydrolytic stability, as the weight loss of the nanofiber mats decreased from ~90% in the non-crosslinked samples to less than 10% after 2 h of crosslinking. Dynamic mechanical analysis (DMA) showed an increase in Young’s modulus from ~70 MPa to 230 MPa after crosslinking. Overall, the results demonstrate the stabilizing effect of citric-acid crosslinking on PVA/HA nanofibers and support their potential use in wound dressings under physiological conditions.

## 1. Introduction

Nanostructured mats assembled from electrospun polymeric nanofibers are an innovative alternative for wound care applications with respect to traditional materials, as they offer several advantages that make them particularly suited for this purpose [1,2]. The nanofibers’ three-dimensional structure closely reproduces the morphology of the natural extracellular matrix (ECM), facilitating cells migration and tissue proliferation, which are vital for a fast and complete healing process [3]. Nanofiber mats possess a large surface area providing good absorption of the exudate, which helps to maintain the cleanliness of the wound and prevents the occurrence of infections [4]. Indeed, the dressing ability to take in the exudate is crucial in the initial stages of the healing when the wound is most susceptible to collection of fluids and debris [5].

Polymer nanofibers (NFs) can be engineered to be bioactive, loaded with therapeutic agents, such as antibiotics or growth factors, and can release these agents gradually into the wound, further improving the healing process [6,7]. For example, Mohiti-Asli et al. [8] successfully incorporated ibuprofen into polylactic acid (PLA) nanofibers to create scaffolds for the treatment of acute and chronic wounds. The in vivo evaluation of PLA nanofibrous scaffolds containing 20 wt% ibuprofen confirmed their biocompatibility, and wound coverage with ibuprofen-loaded acellular scaffolds favorably reduced wound contraction. Mahmoudi et al. [9] developed a biocompatible electrospun nanofiber scaffold containing polyvinyl alcohol (PVA), 70S30C bioactive glass, Ag nanoparticles and curcumin. They demonstrated antibacterial activity against *Escherichia coli* and *Staphylococcus aureus.* Gruppuso et al. [10] developed multilayer antibacterial electrospun matrices based on polycaprolactone (PCL), hyaluronic acid (HA), and lactose-modified chitosan. The matrices exhibited antibacterial activity when rifampin was loaded within the PCL layer. In parallel, tests with human skin fibroblasts confirmed optimal biocompatibility and bioactivity.

Among the broad spectrum of polymers available for nanofiber production, polyvinyl alcohol (PVA) and hyaluronic acid (HA) are the most widely used in wound care [11,12,13]. PVA is a synthetic polymer used for the production of nanofibers for wound healing due to its biocompatibility, biodegradability, water solubility, and high mechanical strength [14]. Furthermore, PVA stands out for the straightforward control over nanofiber morphology and size during electrospinning, and it can be processed in water, thus avoiding the use of organic solvents that may have toxic effects [15]. This versatility of PVA provides a wide range of choices for making nanofiber dressings with tunable properties for specific wound care needs. Besides biocompatibility and mechanical strength, PVA is known to have high water solubility. This capability allows for the incorporation of therapeutic agents or other hydrophilic molecules into the matrix of nanofibers, thus enhancing the effectiveness of the dressing in promoting wound healing. For example, hyaluronic acid (HA), a natural polysaccharide found in the ECM of connective tissues such as skin [16], can be added to the electrospinning solution during the nanofiber matrix fabrication. The chemical composition of HA, consisting of repeating units of glucuronic acid and N-acetylglucosamine, gives it unique properties relevant to the wound healing process. HA exhibits a high capacity to bind and retain water molecules [17], thus providing a moist microenvironment that supports cell migration, proliferation, and angiogenesis. These are all essential processes for effective wound healing [18]. In addition, the viscoelastic properties of HA contribute to tissue lubrication and provide an environment suitable for biological processes, such as cellular activities that are necessary for tissue regeneration [19]. Besides its moisture-storing characteristic, HA plays a crucial role not only in regulating inflammatory responses but also in the process of wound healing. In fact, it acts by regulating leukocyte recruitment, cytokine production, and matrix metalloproteinase, thereby fostering an inflammatory environment conducive to optimal healing outcomes [20]. In addition, HA stimulates angiogenesis, which accelerates the healing process associated with the growth of new blood vessels, an essential activity for nutrient supply and waste removal in the wound bed [21]. The novelty of this work lies in the implementation of a sustainable solid-state esterification strategy relying on biocompatible citric acid integrated directly in the electrospun PVA/HA nanofibers. This solid-state, citric-acid-mediated esterification strategy avoids solution-phase crosslinking and the use of potentially toxic chemical crosslinkers, while maintaining a fully aqueous processing route. The proposed method facilitates a systematic evaluation of how varying thermal treatment times influence the crystallinity, thermal behavior, swelling resistance, and degradation stability of the electrospun mats. Unlike previous studies relying on solution-phase crosslinking or PVA-only systems, the methodology proposed here provides an environmentally sustainable and application-oriented route for producing mechanically stable PVA/HA nano-fibers suitable for wound-healing and other biomedical uses.

In this work, PVA/HA nanofiber mats based on biocompatible polymers are fabricated by the electrospinning technique starting from fully aqueous solutions, so to avoid the use of organic solvents whose residues may have negative effects on wound healing. The process parameters of the electrospinning are optimized to obtain fibers of nanometer size and regular shape. Citric acid (CA) is incorporated into the nanofibers to improve their stability in the physiological wound environment. Specifically, PVA/HA/CA fibers are chemically crosslinked by catalyst-free solid-state esterification, which improved their stability and performance. Scanning electron microscopy (SEM) is used to evaluate the size and morphology of the NF mats. The nanofiber chemical composition is examined using FTIR spectroscopy, and differential scanning calorimetry (DSC) is used to assess the thermal behavior. We present a detailed study of the swelling kinetics of PVA/CA and PVA/HA/CA nanofibers crosslinked using different thermal treatments. Tests are performed at physiological conditions (37 °C) at different pH values (7.4, 8, 9) to evaluate the ability of the NF mats to absorb fluids under conditions mimicking the wound environment. Finally, in vitro degradation tests to evaluate the stability of the nanofibers after chemical crosslinking are reported.

## 2. Materials and Methods

### 2.1. Materials

PVA with a molecular weight of 130 kDa and 86.7–88.7% degree of hydrolysis and anhydrous citric acid were from Sigma-Aldrich (Merck Life Science S.r.l., Milan, Italy) and used as received. Hyaluronic acid sodium salt with a molecular weight of 1500–1800 kDa was purchased from ACEF (Chemical and Pharmaceutical Company S.p.A, Piacenza, Italy). Deionized water (resistivity 18.2 MΩ·cm) was produced by a Direct-Q3 UV water purification system (Merck Millipore S.p.A., Milan, Italy) and used in all preparations.

### 2.2. Fabrication of Nanofiber Mats

**Figure 1:**
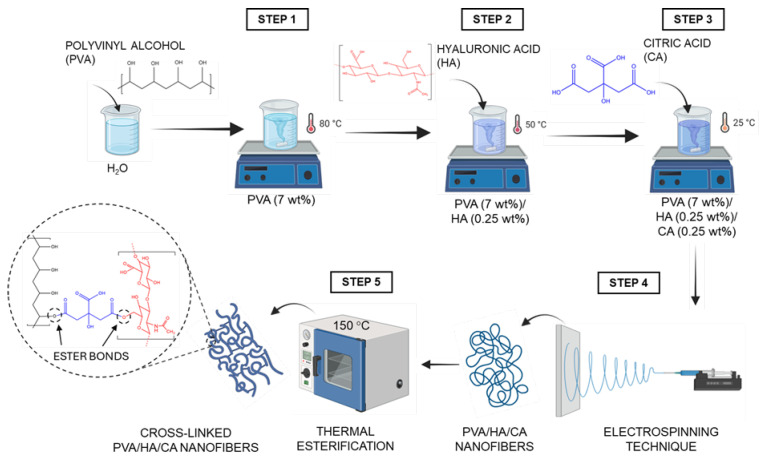
Schematic representation of the nanofiber fabrication process, involving electrospinning of water-based solutions containing PVA, hyaluronic acid (HA) and citric acid (CA), followed by thermal esterification crosslinking at 150 °C. Created in Biorender.Gianluca Ciarleglio (2025).

The nanofiber mats were fabricated using the electrospinning technique following several steps. First, PVA (7–10 wt%) was dissolved in ultrapure water at 80 °C using a magnetic stirrer (C-MAG HS7, IKA, Staufen, Germany) for 1 h. HA (0.25–1 wt%) was then incorporated into the solution at 50 °C and stirred for 1 h. Next, the solution was cooled to room temperature (25 °C) and CA (0.25–1 wt%) was added, and the final mixture stirred for 30 min. For electrospinning, the solution was loaded into a syringe and pumped at a flow rate of 0.5 mL/h through a stainless-steel nozzle with an inner diameter of 0.514 mm (21 G) using an infusion pump (NE-300, New Era Pump Systems, Farmingdale, NY, USA). The spinneret was connected to a high-voltage power supply (CM5, Simco-Ion, Lochem, The Netherlands) at 20–30 kV. The nanofibers formed at the tip of the spinneret were deposited on an aluminum foil. The collecting distance was set at 10 cm. All electrospinning experiments were carried out inside a controlled enclosure to ensure stable ambient conditions, maintaining a temperature of 25 °C and relative humidity (RH) below 50% [22]. Once the nanofibers were obtained, they were subjected to a thermal treatment at 150 °C for various times: 0.5, 1 and 2 h. The purpose of this treatment is to activate the formation of ester bonds between the carboxyl groups of CA and the hydroxyl groups of PVA and HA, thus chemically crosslinking the polymer network. Figure 1 shows a schematic representation of the fabrication process of the crosslinked nanofibers, including solution preparation, electrospinning, and thermal esterification.

### 2.3. Characterization Methods

#### 2.3.1. Morphological Characterization

The morphology and microstructural features of the nanofibers were examined by scanning electron microscopy (SEM) using a VEGA II LSH instrument (Tescan, Brno, Czech Republic). Images were acquired at an accelerating voltage of 10 kV at magnifications of 10,000× and 20,000×. Before imaging, the nanofibers were coated with gold using the Cressington 108 auto sputter coater (Cressington Scientific Instruments, Watford, UK). Image analysis was carried out using ImageJ (v.1.51w) in combination with the DiameterJ plugin (v.1.018) to obtain quantitative measurements of fiber diameter [23]. The following pre-processing steps were carried out: each image was converted to greyscale (8-bit) and saved in TIFF format with ImageJ. Before processing, each micrograph was converted to 8-bit grayscale and saved in TIFF format, and segmentation was performed through DiameterJ to identify fibers and reduce background noise [24].

#### 2.3.2. Thermal Analysis

The thermal properties of the nanofiber mates were investigated using a DSC 8500 calorimeter (PerkinElmer, Waltham, MA, USA). Each measurement was performed on ~10 mg of sample, sealed in aluminum pans and heated under a 40 mL/min nitrogen flow. For each sample type, three independent samples were analyzed. PVA, CA, HA powders, and the various types of fabricated nanofiber mats were investigated in the temperature range from 25 to 250 °C, at heating rates of 10 °C/min. The glass transition temperature (T_g_) was determined from each DSC thermogram by the inflection point method, according to the ASTM E1356-08 standard [25], using the thermal analysis software (PerkinElmer, Waltham, MA, USA) provided with the instrument.

#### 2.3.3. FTIR Spectroscopy

The chemical structures of PVA, HA, and CA powders and the various types of NF mats were investigated using a Nicolet Summit FTIR Spectrometer (Thermo Fisher Scientific, Waltham, MA, USA) in attenuated total reflectance (ATR) mode. Each spectrum was acquired from 64 scans co-added at a spectral resolution of 4 cm^−1^, in the wavenumber range of 800–4000 cm^−1^. All FTIR spectra were processed using OMNIC Paradigm software v. 2.0 (Thermo Fisher Scientific, Waltham, MA, USA), applying ATR correction to account for ATR-related intensity and band position distortions, followed by automated baseline correction to ensure consistent baseline alignment prior to peak analysis. The crystallinity of the nanofibers was assessed by deconvolution of the spectra. OriginPro 2024 (OriginLab Corporation, Northampton, MA, USA) was used for peak deconvolution with Gaussian fitting (R^2^ > 0.99), applying a quadratic Savitzky–Golay filter for derivative smoothing. The crystallinity index (CI) was calculated using the following formula [26]:

(1) $$CI=\frac{I_{1140}}{I_{1090}}\times 100$$

where I_1140_ is the intensity of the peak associated with the crystalline phase of NFs, corresponding to the symmetric stretching of the C-O-C group, and I_1090_ is the intensity associated with the peak chosen as the reference [27].

#### 2.3.4. Polymer Solution Properties

The electrospinning solutions were analyzed in terms of viscosity and electrical conductivity. Viscosity was determined using a micro-Ubbelohde capillary viscometer (Xylem Analytics Mainz, Germany). The viscosity of PVA, PVA/CA, PVA/HA, and PVA/HA/CA solutions was consistently evaluated at a controlled temperature of 25 °C. For each measurement, 3 mL of solution was introduced into the viscometer. Viscosity was calculated based on the flow time of the liquid meniscus of the solution from the upper indicator (M1) to the lower indicator (M2) on the viscometer. The flow time detected by the viscometer, expressed in seconds, was then multiplied by the capillary constant (k = 0.9935 mm^2^/s) to obtain the kinematic viscosity expressed in CentiStokes (cSt). Conductivity values of solutions were recorded using a conductivity meter (Mettler Toledo, Five Go F3, Schwerzenbach, Switzerland), with a conductivity electrode (cell constant 0.554 cm^–1^) made of polycarbonate. The measurement was repeated three times per sample. The temperature was maintained at 25 °C throughout the experiment.

#### 2.3.5. Swelling Behavior

To determine the swelling kinetics, the different types of nanofiber mats (~10 mg) were first completely dried at 50 °C for 18 h and then immersed in 15 mL of phosphate-buffered saline (PBS) solution at various pH values. The pH values chosen for this test are 7.4, 8, and 9, as they represent and mimic the microenvironment of the wound [28,29,30]. The kinetics were evaluated for 48 h at the physiological temperature of 37 °C. The swelling ratio was calculated with the following equation [31,32,33,34]:

(2) $$Swelling\;Ratio=\frac{W_{s}}{W_{d}}$$

where W_s_ is the weight of the swollen sample at different times and W_d_ is the weight of the fully dried sample. The behavior of the nanofibers was monitored every hour for the first 5 h, and then after 24 h and 48 h. The test was repeated on 5 samples for each type of NFs.

#### 2.3.6. In Vitro Degradation Tests

In vitro degradation tests were conducted on PVA/CA and PVA/HA/CA nanofiber mats with thermal treatment of 0.5, 1, and 2 h (5 samples for each measurement). 10 mg samples were weighed and placed in glass vials. Subsequently, 15 mL of PBS buffer solutions at different pH values (7.4, 8, 9) were added. The test was carried out using a thermostatic bath to simulate the body temperature of 37 °C. After 48 h, these samples were extracted from the solution and dried in an oven at 50 °C for 18 h. Finally, the NF mats were weighed, and weight loss was assessed using the following formula [35]:

(3) $$Weight\;loss\left(\%\right)=\frac{W_{i}-W_{f}}{W_{i}}\times 100$$

where W_f_ and W_i_ are the final and the initial weights of the sample after 48 h of degradation, respectively.

#### 2.3.7. Mechanical Characterization

Quasi-static tensile tests were carried out using a DMA1 Dynamic Mechanical Analyzer (Mettler Toledo, Greifensee, Switzerland). All measurements were performed in air at a controlled temperature of 25 °C. The applied load was increased from 0 to 5 N at a constant rate of 0.1 N/min, and uniaxial tensile tests were used to obtain the corresponding stress–strain curves. For each sample type, three independent specimens with dimensions of 5 mm in length, 5 mm in width, and a thickness between 80 and 100 µm were analyzed. The Young’s modulus was calculated by applying a linear fit to the initial elastic region of the stress–strain response, specifically within the axial strain range of 0–1%.

## 3. Results

### 3.1. Fabrication and Optimization of Nanofibers

Preliminary tests on the fabrication of PVA-based nanofibers were conducted to optimize the process parameters of the electrospinning technique. Specifically, the PVA solution was initially fixed at 10 wt%, and the adopted process parameters for the electrospinning were a needle size of 21 G, a flow rate of 0.5 mL/h, a collection distance of 10 cm, and a voltage ranging from 15 to 30 kV. Next, HA was incorporated into the PVA fibers. Different concentrations of HA, in the range of 0.25 to 1 wt%, were tested to obtain an electrospinnable polymer solution. Different concentrations of HA, in the range of 0.25 to 1 wt%, were tested to obtain an electrospinnable polymer solution. At 0.5 and 1 wt% HA no stable jet was formed due to excessive viscosity. Therefore, a lower PVA concentration of 7 wt% was adopted. After obtaining the PVA fibers and adjusting the process parameters, HA was incorporated. In this case, HA concentrations of 0.25 and 0.5 wt% were tested, but stable nanofibers were achieved only with 0.25 wt% HA combined with 7 wt% PVA.

Table 1 summarizes the combinations of PVA, HA and CA tested, showing that nanofibers were obtained at 7 wt% PVA with 0.25 wt% HA, while higher HA contents prevented fiber formation.

Citric acid was chosen to crosslink the polymer network by esterification through thermal treatments. CA was introduced into the PVA/HA fibers at 0.25, 0.5 and 1 wt%. Continuous nanofibers were obtained only with 0.25 wt% CA, while higher concentrations prevented fiber formation.

### 3.2. SEM Morphological Analysis

Preliminary morphological investigations were performed to assess the effect of electrospinning voltage and solution composition on fiber formation. Figure S1 shows SEM images of PVA nanofibers (10 wt%) electrospun at voltages ranging from 15 to 30 kV. Increasing the applied voltage resulted in a progressive reduction in the mean fiber diameter, from ~186 nm at 15 kV to ~150 nm at 30 kV, consistent with enhanced electrohydrodynamic stretching [36]. However, at higher voltages (25–30 kV) bead-like defects became apparent, indicative of jet instabilities [37]. Diameter distributions (Figure S1c,f,i,l) confirmed this trend, with broader distributions and a tail toward finer diameters at higher voltages. Quantitative analysis (Table 2) showed that porosity remained nearly constant across voltages, whereas intersection density increased with voltage, suggesting enhanced fiber interconnection. These findings demonstrate that applied voltage influences both fiber size and network architecture, balancing stretching forces and jet stability.

**Figure 2:**
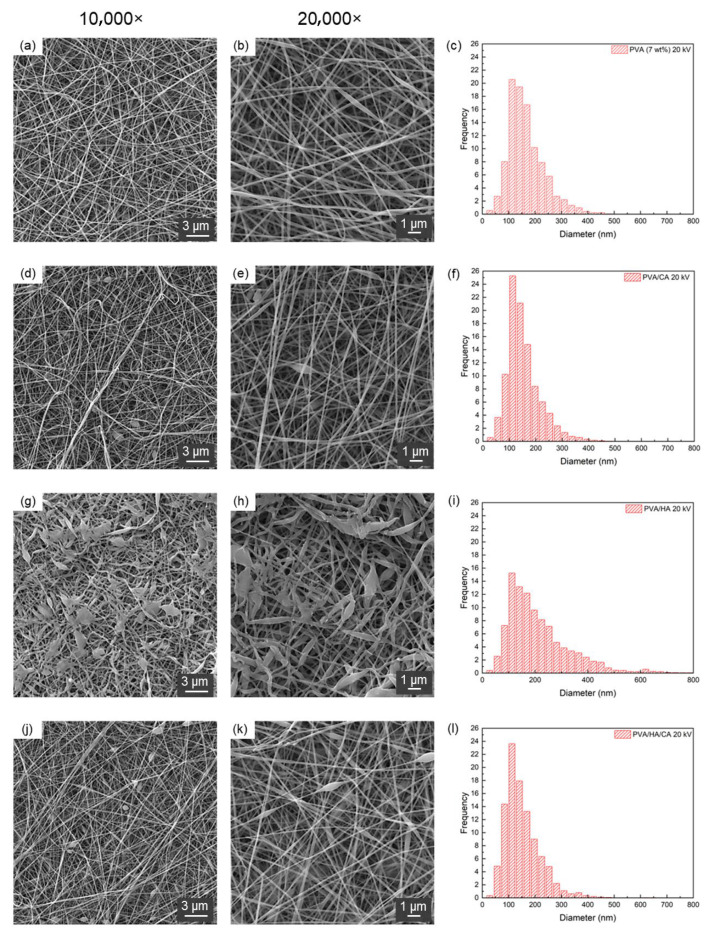
SEM images acquired at (**a**,**d**,**g**,**j**) 10,000× and at (**b**,**e**,**h**,**k**) 20,000× and (**c**,**f**,**i**,**l**) nanofiber diameter distribution of (**a**–**c**) PVA, (**d**–**f**) PVA/CA, (**g**–**i**) PVA/HA and (**j**–**l**) PVA/HA/CA. The nanofibers were fabricated using a voltage of 20 kV.

Figure 2 shows the SEM images and diameter distributions of PVA, PVA/CA, PVA/HA and PVA/HA/CA nanofibers electrospun at 20 kV. Neat PVA and PVA/CA fibers (Figure 2a–f) were smooth, uniform and bead-free, with mean diameters below 200 nm and relatively narrow size distributions. The incorporation of HA (Figure 2g–i) led to flattened fibers and bead formation, attributable to the high viscosity of the HA-containing solution, which slowed solvent evaporation and promoted fiber collapse upon deposition. When HA and CA were combined (Figure 2j–l), fibers exhibited intermediate features: thinner diameters than PVA/HA, improved interconnection and reduced bead density.

Table 3 summarizes the quantitative analysis of fiber morphology and solution properties. All formulations produced nanometric diameters. Among the fabricated fibers, PVA/HA/CA fibers exhibit the highest kurtosis (3.60), followed by PVA/HA (2.87), PVA/CA (2.28), and finally PVA (2.04). This indicates that PVA/HA/CA fibers have a more leptokurtic diameter distribution compared to other types. Kurtosis was included as an additional descriptor to assess the sharpness of the diameter distribution and the presence of heavy tails, complementing mean diameter and standard deviation in evaluating nanofiber uniformity.

These results show that the PVA/CA fibers have the highest intersection density (4.65 ints/μm^2^), followed by PVA/HA/CA (4.43 ints/μm^2^), PVA (4.30 ints/μm^2^), and PVA/HA (3.45 ints/μm^2^). This suggests that the addition of CA can increase the intersection density of PVA fibers. On the other hand, the results indicate that the addition of HA contributes significantly increases material’ viscosity: PVA/HA fibers show the highest dynamic viscosity (814.1 mPa·s), followed by PVA/HA/CA fibers (393.9 mPa·s), PVA fibers (81.2 mPa·s), and PVA/CA fibers (86.5 mPa·s). From a morphological point of view, the increase in solution viscosity leads to a change in fiber structure: from smooth and uniform fibers (Figure 2a–d) to flat fibers with an increased presence of beads (Figure 2e–h). This phenomenon can be attributed to the fact that a more viscous solution slows solvent evaporation, allowing the wet fibers to flatten upon impact with the collector [38]. The electrical conductivity of the samples increased with the addition of all additives, with the highest value observed for the HA/CA combination (770.6 ± 0.8 μS/cm), followed by PVA/CA (620.7 ± 0.7 μS/cm) and PVA/HA (511.8 ± 1.5 μS/cm), in comparison to the pure PVA (301.9 ± 1.5 μS/cm). The increase in electrical conductivity observed in samples with additives can be explained based on the intrinsic properties of these additives. Hyaluronic acid (HA) and citric acid (CA) contain ionizable groups that increase the number of charge carriers, facilitating the movement of electrical charges through the polymers matrix [39]. Overall, the addition of CA tends to reduce the diameter and increase the porosity and the electrical conductivity of the fibers, while HA significantly increases the viscosity and affects the fiber diameter distribution. The combination of HA and CA shows synergistic effects, producing fibers with intermediate characteristics.

### 3.3. Chemical Analysis and Crystallinity by FTIR Spectroscopy

**Figure 3:**
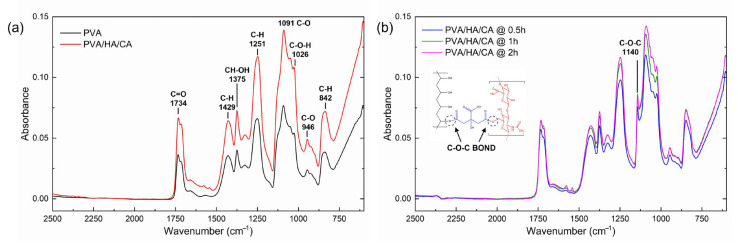
FTIR spectra of electrospun PVA and PVA/HA/CA nanofibers (**a**) without thermal treatment and (**b**) after treatment at 150 °C for different times showing the appearance of the 1140 cm^–1^ band corresponding to the formation of the ester bond.

FTIR analysis was performed to evaluate the chemical composition and covalent bonds environment of the electrospun nanofibers, and to assess the crosslinking esterification process after the thermal treatment at 150 °C. Results are shown in Figure 3. In particular, Figure 3b shows a comparison of the spectra of the PVA/HA/CA nanofibers that were treated at 150 °C for different times (0.5 h, 1 h, 2 h). FTIR analysis was performed to chemically verify the presence of new crosslinking bonds due to the citric-acid mediated esterification at 150 °C. Compared with the spectra of non-thermally treated PVA and PVA/HA/CA nanofibers (Figure 3a), the thermally treated nanofibers show the appearance of a new peak at 1140 cm^–1^. This peak is attributed to the symmetric stretching of the C–O–C bond, which is characteristic of esters. This result suggests that esterification occurred between PVA and citric acid (CA), as confirmed in the literature [40]. This peak is related to the crystal structure of PVA; its presence indicates an increase in the crystalline phase compared to non-thermally treated PVA and PVA/HA/CA nanofibers [41].

**Figure 4:**
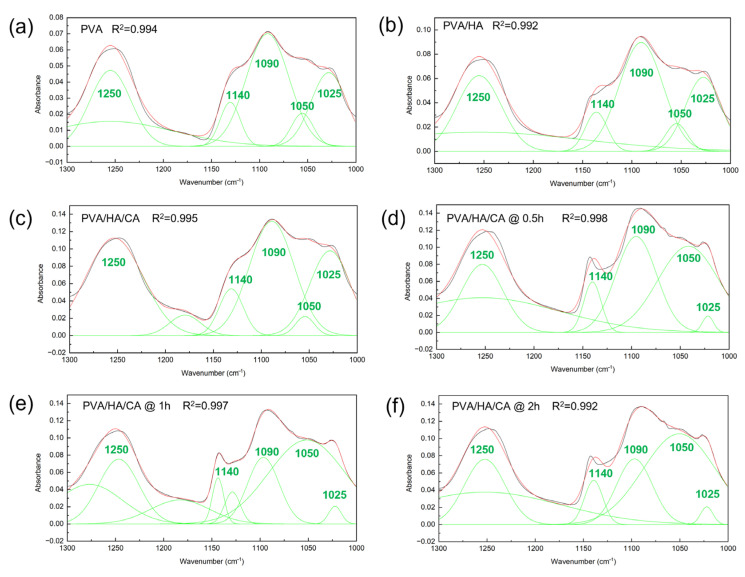
Deconvolution of FTIR spectra in the region from 1300 cm^–1^ to 1000 cm^–1^ for (**a**) PVA, (**b**) PVA/HA, (**c**) PVA/HA/CA, and thermally crosslinked PVA/HA/CA at different times: (**d**) 0.5 h, (**e**) 1 h, (**f**) 2 h. The black curve is the experimental FTIR spectrum, the red curve the global fitted curve, while the green curves correspond to the individual Gaussian components used for the deconvolution.

The crystallinity index of the NFs was evaluated by deconvolution of the FTIR spectra, which allowed the calculation of the ratio between the peak at 1140 cm^–1^, related to the crystalline phase, and the peak at 1090 cm^–1^, the constant. The peak related to the crystalline phase is not easily observed in the 1300–1000 cm^–1^ region because the width of the bands of the different components is greater than the separation between the peaks of each band. The deconvolution of the spectrum was used to identify the hidden peaks as described in the Materials and Methods section. Convergence, defined by reaching the χ^2^ tolerance value of 10^–6^, was obtained for all deconvolution curves. The goodness of fit was evaluated by the parameter R-square > 0.99. The deconvoluted spectra, together with the individual Gaussian components used for the deconvolution, are shown in Figure 4.

Table 4 shows the crystallinity index (CI) values obtained by the FTIR technique for PVA nanofibers combined with HA and CA at different thermal treatment times. The introduction of hyaluronic acid results in a decrease in crystallinity compared to pure PVA, reducing from 42.9 ± 1.6 to 40.3 ± 1.0. This is attributed to the steric hindrance caused by the longer molecular chains of HA. These chains prevent the formation of ordered crystalline structures in the PVA polymer matrix as observed by Zhang et al. [42] An increase in crystallinity is observed in thermally treated nanofibers compared to untreated ones. This phenomenon is due to the chemical crosslinking (esterification) process induced by the thermal treatment, which promotes a more ordered organization of the polymer structure and a denser network [43].

### 3.4. Thermal Properties by DSC

A preliminary DSC analysis was performed on the PVA, CA, and HA powders to evaluate their thermal properties (Figure S2). The thermogram of PVA shows a glass transition at 45.71 ± 0.70 °C, followed by a melting endotherm at 188.93 ± 0.50 °C with a melting enthalpy (ΔH_m_) of 41.54 ± 0.24 J/g. Citric acid exhibits a sharp melting peak at 161.59 ± 0.33 °C, with a corresponding ΔH_m_ of 215.73 ± 0.09 J/g. Hyaluronic acid shows an exothermic event at approximately 235.02 ± 0.63 °C, consistent with the onset of thermal degradation. These data provide a reference for evaluating the thermal behavior of the electrospun nanofibers.

Differential scanning calorimetry was then used to analyze the glass transition temperature (T_g_), the melting temperature (T_m_), and the thermal stability in the physiological range of the nanofiber mats.

**Figure 5:**
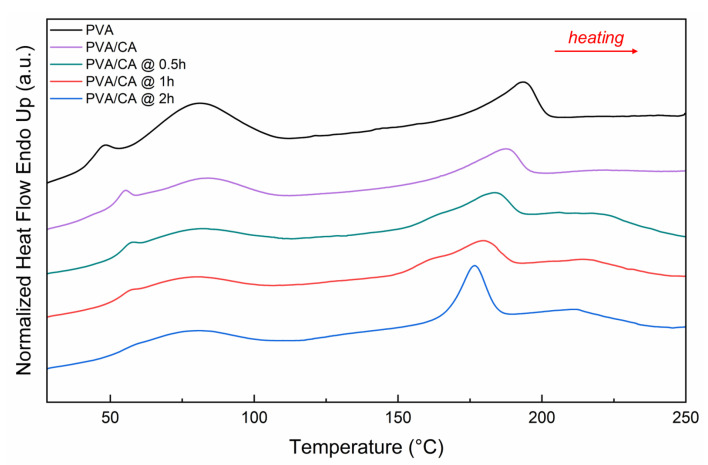
DSC thermograms (heating rate 10 °C/min) of crosslinked PVA, PVA/CA nanofibers with different times (0.5 h, 1 h, 2 h) of thermal treatment.

Figure 5 shows the thermograms of PVA, PVA/CA, and PVA/CA with different crosslinking times. All the thermograms show a peak around 80 °C, indicating the evaporation of water contained in the nanofiber layers. As the crosslinking time increases, the intensity of the water evaporation peak decreases, reflecting the reduced presence of water in the fibers [44].

The intensity of the phenomenon related to T_g_ decreases as crosslinking time increases, as indicated by the values of Δc_p_ (Table 5). The reduction in T_g_ of PVA could be attributed to less water content in the samples due to thermal treatments, which reduces the plasticizing effect of water on the material [45]. In contrast, T_m_ decreases with the addition of citric acid and increasing crosslinking time. The introduction of organic acids can chemically inhibit PVA crystallization due to the strong interaction between free hydrogen and –OH bonds formed with citric acid, resulting in a decrease in T_m_, as reported in the literature [46].

**Figure 6:**
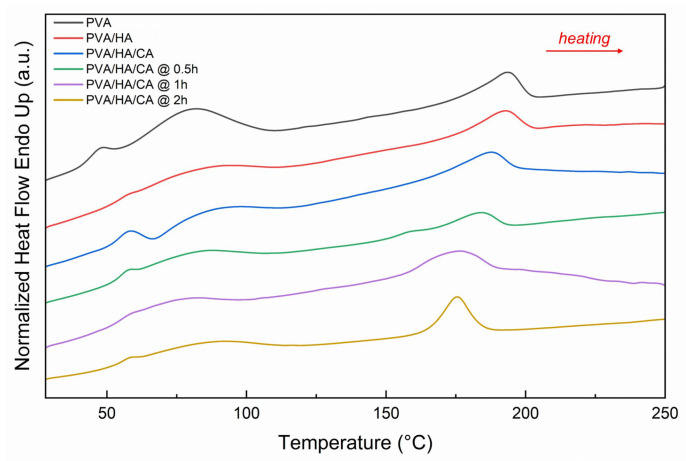
DSC thermograms (heating rate 10 °C/min) of crosslinked PVA, PVA/HA, and PVA/HA/CA nanofibers with different times (0.5 h, 1 h, 2 h) of thermal treatment.

Figure 6 shows the thermogram of PVA, PVA/HA, and PVA/HA/CA at various crosslinking times. Here again, the behavior is similar to the PVA/CA fiber. There is an increase in T_g_ and a decrease in T_m_ with the addition of HA and citric acid at different crosslinking times (Table 6). Compared with PVA/HA (T_g_ = 52.0 °C; T_m_ = 192.5 °C), the presence of CA shifted T_g_ slightly upward (52.9 °C) and T_m_ downward (188.2 °C). With longer crosslinking times, T_g_ reached 55.4 °C after 2 h, while T_m_ decreased to 176.8 °C. The melting enthalpy (ΔH_m_) showed moderate variation, suggesting a partial reduction in crystallinity.

Thermal analysis also showed the stability of the nanofibers in the physiological range (35 °C to 40 °C), making them suitable for wound treatment.

### 3.5. Swelling Kinetics

**Figure 7:**
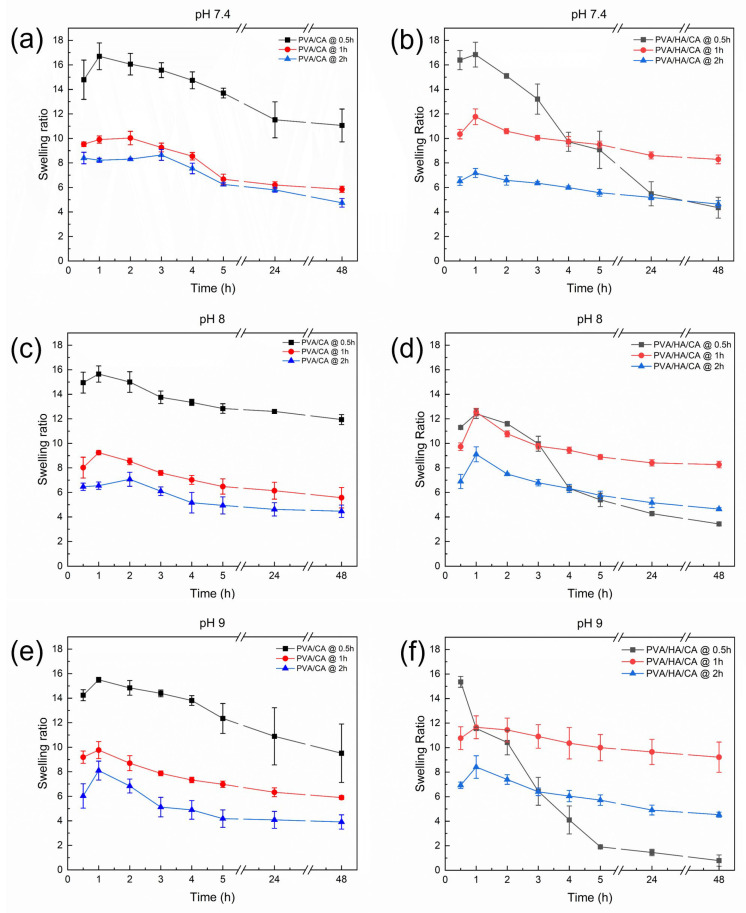
Swelling kinetics of the (**a**,**c**,**e**) PVA/CA and (**b**,**d**,**f**) PVA/HA/CA nanofibers at 37 °C and different pH values: (**a**,**b**) 7.4, (**c**,**d**) 8.0, and (**e**,**f**) 9.0.

The evaluation of swelling kinetics for PVA/CA and PVA/HA/CA nanofibers is crucial as it provides key insights into the samples ability to absorb fluids under conditions that mimic the wound environment. Preliminary tests on non-thermally crosslinked NFs were performed by immersion in PBS. The test showed immediate dissolution of the nanofibers. Swelling kinetics were evaluated for PVA/CA and PVA/HA/CA NFs after crosslinking in an oven at 150 °C for three different times (0.5, 1, 2 h). Tests were performed using PBS solutions at three pH values (7.4, 8, 9) to simulate a wound environment. Figure 7 shows the trend of the swelling ratio as a function of fiber immersion time in PBS, pH, and crosslinking time.

All samples show an initial increase in swelling ratio followed by a decrease over time. This indicates that the fibers initially absorb liquids rapidly, followed by a degradation phase. There is a tendency for the swelling ratio to decrease as the crosslinking time increases. This suggests that a longer crosslinking time leads to greater stability of the polymer structure, which reduces the liquid-absorbing capacity of the fibers. In samples crosslinked for 0.5 h, it is observed that the peak swelling occurs slightly faster at pH 9.0 than at pH 7.4 and 8.0. This may indicate that a more alkaline environment promotes faster absorption of liquids by the fibers. There does not appear to be a significant difference in the swelling values of the fibers at different pH levels. All samples showed a similar trend over time regardless of the pH of the solution. However, the PVA/HA/CA nanofibers thermally crosslinked for only 0.5 h exhibit a distinct behavior compared to the other samples. These fibers did not preserve their structural integrity during the swelling test, showing a rapid decrease in the swelling ratio due to partial dissolution and fragmentation of the mat. This observation suggested that a thermal treatment of 0.5 h was not sufficient to achieve a crosslinking degree of the nanofibers compatible with the application in physiological environment.

### 3.6. Biodegradation Test

Table 7 shows the biodegradability results of chemically crosslinked PVA/CA and PVA/HA/CA NFs mats by thermal treatment at different pH values (7.4, 8.0, 9.0) at 37 °C after 48 h. Preliminary tests were conducted on the non-crosslinked PVA/CA and PVA/HA/CA nanofibers. The tests showed that the non-crosslinked nanofibers dissolved instantly, confirming the importance of chemical crosslinking in improving the stability of the nanofibers.

**Figure 8:**
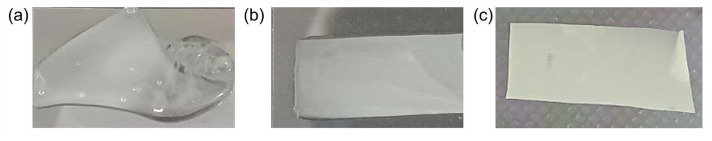
(**a**) PVA/HA/CA @ 0.5 h, (**b**) PVA/HA/CA @ 1 h, and (**c**) PVA/HA/CA @ 2 h nanofibers after 2 h of immersion in PBS solution at pH 8.

There is a general tendency for greater weight loss in PVA/HA/CA fibers than in PVA/CA fibers. This suggests that the presence of HA affects the stability of the fiber, resulting in greater degradation. This phenomenon may be related to the previously observed crystallinity values. PVA/HA/CA nanofibers have lower crystallinity than PVA/CA nanofibers. The lower crystallinity indicates a greater amorphous arrangement in the nanofiber structure, which in turn may promote greater accessibility of ester bonds to the hydrolysis process. As a result, fibers with lower crystallinity tend to degrade more than those with higher crystallinity. For both types of fibers, a decrease in weight loss is observed with increasing crosslinking time. In the case of PVA/CA @ 0.5 h, a significantly greater weight loss is observed compared to PVA/CA @ 1 h and PVA/CA @ 2 h. Figure 8 displays the appearance of PVA/HA/CA nanofibers realized with different crosslinking times (0.5, 1, 2 h) after 2 h of immersion in PBS solution at pH 8. The results suggest that a longer crosslinking time leads to greater fiber stability and consequently, less degradation. There is no clear trend observed regarding the effect of pH on weight loss. Overall, these findings suggest that both crosslinking time and chemical composition of the fibers significantly influence their stability and degradability.

### 3.7. Quasi-Static Tensile Testing

**Figure 9:**
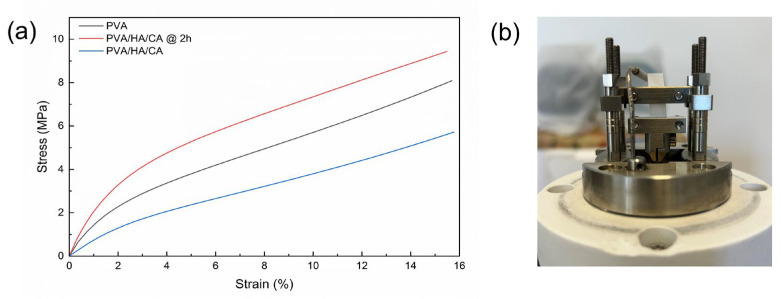
(**a**) Stress–strain curves of PVA, PVA/HA/CA, and PVA/HA/CA @ 2 h. Curves are obtained from quasi-static tensile tests by DMA. (**b**) DMA measurement configuration.

The stress–strain curves of PVA, PVA/HA/CA, and PVA/HA/CA @ 2 h are shown in Figure 9. Results show that the nanofibers can withstand a 16% strain without fracturing, indicating their high ductility and good elongation capacity. This means the material can undergo significant deformation under stress without compromising its structural integrity.

For the determination of the Young’s modulus, the nominal axial stress versus the axial strain data were fitted linearly in the range from 0 to 1% and the slope was calculated. The linear fitting achieved an R-square > 0.99 for all sample types, indicating high correlation and reliability in determining the longitudinal elastic modulus. The calculated longitudinal elastic modulus values for the various samples are summarized in Table 8.

Results highlight the differences in the mechanical properties of the wound dressings. The PVA/HA/CA @ 2 h sample exhibited the highest elastic modulus, indicating it is stiffer than the other samples. This significant increase in stiffness is likely due to the thermal treatment at 150 °C for 2 h, which promotes chemical crosslinking through esterification between the polyvinyl alcohol and citric acid. This crosslinking restricts chain mobility, leading to a higher elastic modulus. The PVA/HA/CA sample, without thermal treatment, shows a lower modulus, suggesting that the absence of crosslinking results in a more compliant material. The PVA sample, though higher in modulus than PVA/HA/CA, is still less stiff compared to the thermally treated PVA/HA/CA @ 2 h, highlighting the critical role of the esterification process in enhancing the material properties. Human skin exhibits highly variable mechanical properties depending on anatomical site, hydration, and testing methodology, with reported Young’s modulus values generally ranging between 0.05 and 20 MPa and exceeding 100 MPa in specific layers or loading conditions [47,48]. Recent uniaxial tensile measurements on human dermis showed moduli of ~0.10–20.6 MPa [49]. In comparison, the fully crosslinked PVA/HA/CA nanofiber mats in our study display a Young’s modulus of ~238 MPa in the dry state, which is consistent with values typically reported for dry electrospun polymers. A substantial decrease in stiffness is expected upon hydration; however, mechanical testing under physiologically relevant conditions will be required to determine how closely the hydrated fibers approach the mechanical range of wound tissues.

## 4. Conclusions

Nanofibers based on PVA and hyaluronic acid, crosslinked with citric acid, were successfully produced by electrospinning without the use of organic solvents. The chemical crosslinking achieved by thermal treatments, without chemical catalysts, represents a simpler and safer method of enhancing nanofiber stability. Dimensional analysis confirmed that the fibers are nanometer-size, with diameters less than 200 nm. Morphological analysis showed that the viscosity of the solution affected the formation of imperfections in the nanofibers. FTIR spectroscopic analysis confirmed the crosslinking of the nanofibers by the esterification process, while DSC analysis assessed the thermal behavior of the nanofibers and demonstrated their stability within the physiological range. Studies on swelling kinetics and biodegradability showed that fiber stability improves with longer crosslinking time. In addition, the increase in crystallinity of the nanofibers after thermal treatment suggests the formation of a denser polymer network. These results highlight the potential of engineered nanofibers for biomedical applications, particularly in the context of tissue regeneration for wound care, offering promising prospects in the field of regenerative medicine. However, further in vitro and in vivo studies will be required to fully evaluate their biocompatibility, biological performance, and clinical translation potential.

## Figures and Tables

**Table 1:** Summary of electrospinning parameters for NFs production.

PVA (wt%)	HA (wt%)	CA (wt%)	Voltage (kV)	Comments
10	0	0	15–30	Successfully electrospun nanofibers
10	1	0	20–30	Fibers not formed
10	0.5	0	20–30	Fibers not formed
7	0	0	20	Successfully electrospun nanofibers
7	0.5	0	20	Fibers not formed
7	0.25	0	20	Successfully electrospun nanofibers
7	0	0.25	20	Successfully electrospun nanofibers
7	0.25	1	20	Fibers not formed
7	0.25	1	25	Fibers not formed
7	0.25	0.5	20	Fibers not formed
7	0.25	0.25	20	Successfully electrospun nanofibers

**Table 2:** Effect of voltage on morphological characteristics of PVA NFs with a concentration of 10 wt%. The table shows the mean diameter, diameter kurtosis, porosity percentage, and intersection density of electrospun PVA nanofibers at different voltages (15 kV, 20 kV, 25 kV, and 30 kV).

	PVA 15 kV	PVA 20 kV	PVA 25 kV	PVA 30 kV
Mean Diameter(nm)	185.7 ± 62.6	183.3 ± 62.3	164.6 ± 63.4	149.5 ± 60.2
Diameter Kurtosis	0.74	1.24	1.18	0.83
% Porosity (%)	51.48	49.16	49.45	48.89
Intersection Density (ints/μm^2^)	2.90	3.26	3.85	4.75

**Table 3:** Comparative analysis of morphological and rheological characteristics of PVA (7 wt%) nanofibers electrospun at 20 kV. The table presents the mean diameter, diameter kurtosis, porosity percentage, intersection density, and dynamic viscosity (η) of PVA, PVA/HA, PVA/CA, and PVA/HA/CA NFs.

	PVA	PVA/CA	PVA/HA	PVA/HA/CA
Mean diameter (nm)	152.7 ± 54.4	133.3 ± 43.7	164.3 ± 77.7	130.2 ± 50.3
Diameter kurtosis	2.04	2.28	2.87	3.60
Porosity (%)	48.31	52.87	45.27	54.37
Intersection density (ints/μm^2^)	4.30	4.65	3.45	4.43
Dynamic viscosity (mPa·s)	81.2	86.5	814.1	393.9
Electrical conductivity (μS/cm)	301.9 ± 1.5	620.7 ± 0.7	511.8 ± 1.5	770.6 ± 0.8

**Table 4:** Crystallinity index (CI) of the electrospun NFs as determined by FTIR spectra after deconvolution.

Crosslinking Time	CI (FTIR) (%)			
	PVA	PVA/HA	PVA/CA	PVA/HA/CA
t = 0 h	42.9 ± 1.6	40.3 ± 1.0	45.1 ± 1.1	41.8 ± 0.6
t = 0.5 h	-	-	48.8 ± 1.5	48.4 ± 0.4
t = 1 h	-	-	54.8 ± 0.3	46.6 ± 0.1
t = 2 h	-	-	48.3 ± 0.4	47.4 ± 0.9

**Table 5:** Thermal properties of PVA, PVA/CA, and crosslinked PVA/CA nanofibers as measured by DSC under nitrogen flow (40 mL/min). Values are reported as mean ± standard deviation (*n* = 3).

Sample	T_g_ (°C)	Δc_p_ (J/g × °C)	T_m_ (°C)	ΔH_m_ (J/g)
PVA	41.36 ± 1.65	0.48 ± 0.08	194.02 ± 0.36	36.57 ± 1.48
PVA/CA	48.78 ± 1.86	0.43 ± 0.21	186.48 ± 0.81	36.88 ± 1.58
PVA/CA @ 0.5 h	52.32 ± 0.24	0.52 ± 0.05	182.13 ± 0.61	35.35 ± 0.68
PVA/CA @ 1 h	52.48 ± 0.38	0.28 ± 0.06	179.04 ± 0.30	37.72 ± 1.73
PVA/CA @ 2 h	53.00 ± 1.10	0.16 ± 0.03	177.39 ± 0.80	39.15 ± 1.58

**Table 6:** Thermal properties of PVA/HA, PVA/HA/CA, and crosslinked PVA/HA/CA nanofibers as measured by DSC under nitrogen flow (40 mL/min). Values are reported as mean ± standard deviation (*n* = 3).

Sample	T_g_ (°C)	Δc_p_ (J/g × °C)	T_m_ (°C)	ΔH_m_ (J/g)
PVA/HA	52.03 ± 0.58	0.27 ± 0.05	192.46 ± 0.47	37.99 ± 1.98
PVA/HA/CA	52.92 ± 1.36	0.42 ± 0.08	188.24 ± 0.82	30.91 ± 1.22
PVA//HACA @ 0.5 h	53.89 ± 0.43	0.49 ± 0.05	183.30 ± 0.31	37.41 ± 2.41
PVA/HA/CA @ 1 h	53.81 ± 0.68	0.38 ± 0.02	178.22 ± 1.35	34.41 ± 2.90
PVA/HA/CA @ 2 h	55.43 ± 1.12	0.29 ± 0.03	176.78 ± 0.46	37.71 ± 1.38

**Table 7:** Weight loss (%) of crosslinked PVA/CA and PVA/HA/CA nanofibers with different crosslinking times (0.5, 1, 2 h) evaluated in PBS solution with different pH values (7.4, 8, 9) after 48 h. Values are reported as mean ± standard deviation (*n* = 5).

Sample	Weight Loss (%) After 48 h		
	PBS pH 7.4	PBS pH 8	PBS pH 9
PVA/CA @ 0.5 h	64.80 ± 3.05	61.37 ± 11.30	55.5 ± 7.57
PVA/CA @ 1 h	3.17 ± 0.81	2.77 ± 1.48	2.32 ± 0.27
PVA/CA @ 2 h	1.34 ± 0.59	0.25 ± 0.12	2.97 ± 0.45
PVA/HA/CA @ 0.5 h	85.32 ± 4.03	87.95 ± 3.07	95.04 ± 2.70
PVA/HA/CA @ 1 h	12.63 ± 3.66	8.85 ± 1.13	23.04 ± 2.69
PVA/HA/CA @ 2 h	2.96 ± 0.45	5.92 ± 2.27	3.06 ± 0.90

**Table 8:** Young’s modulus and standard deviation of the PVA, PVA/HA/CA, and PVA/HA/CA @2 h samples. Mechanical properties are expressed as mean ± standard deviation (*n* = 3).

Sample	Young’s Modulus (MPa)
PVA	152.13 ± 27.76
PVA/HA/CA	72.73 ± 7.45
PVA/HA/CA @ 2 h	237.96 ± 14.66

## Data Availability

The original contributions presented in this study are included in the article/Supplementary Material. Further inquiries can be directed to the corresponding author.

## Supplementary Materials

The following supporting information can be downloaded at: https://www.mdpi.com/article/10.3390/polym18010079/s1. Figure S1: SEM images of PVA nanofibers electrospun from 10 wt% polymer solutions at applied voltages of (a,b) 15 kV, (d,e) 20 kV, (g,h) 25 kV, and (j,k) 30 kV. Images acquired at magnifications of 10,000× (a,d,g,j) and 20,000× (b,e,h,k). The corresponding fiber diameter distributions are shown in panels (c,f,i,l). Increasing voltage reduced mean fiber diameter, while bead defects became evident at 25–30 kV (red arrows). Figure S2: DSC thermograms of (a) PVA, (b) HA, and (c) CA as supplied by the manufacturer (powder form) at a heating rate of 10 °C/min.
