# Supplementary material for: Poly(Vinyl Alcohol)/Hyaluronic Acid Nanofibers for Biomedical Use Under Physiological Conditions: Electrospinning Fabrication and Stabilization via Solvent-Free Citric Acid Crosslinking
Source: Polymers (Basel). 2025 Dec 27;18(1):79. doi: 10.3390/polym18010079 (PMC12787809; doi:10.3390/polym18010079)
Supplement: Supplementary file 1 [file polymers-18-00079-s001.zip › polymers-3991418-supplementary.pdf]

Supplementary Materials

Poly(Vinyl Alcohol)/Hyaluronic Acid Nanofibers for Biomedical Use Under Physiological Conditions: Electrospinning Fabrication and Stabilization via Solvent-Free Citric Acid Crosslinking

Gianluca Ciarleglio, Nicholas Capuccilli, Elisa Toto and Maria Gabriella Santonicola *

Department of Chemical Engineering Materials Environment, Sapienza University of Rome, Via del Castro Laurenziano 7, 00161 Rome, Italy; gianluca.ciarleglio@uniroma1.it (G.C.); capuccilli.1797862@studenti.uniroma1.it (N.C.); elisa.toto@uniroma1.it (E.T.)

**Correspondence: mariagabriella.santonicola@uniroma1.it (M.G.S.)*

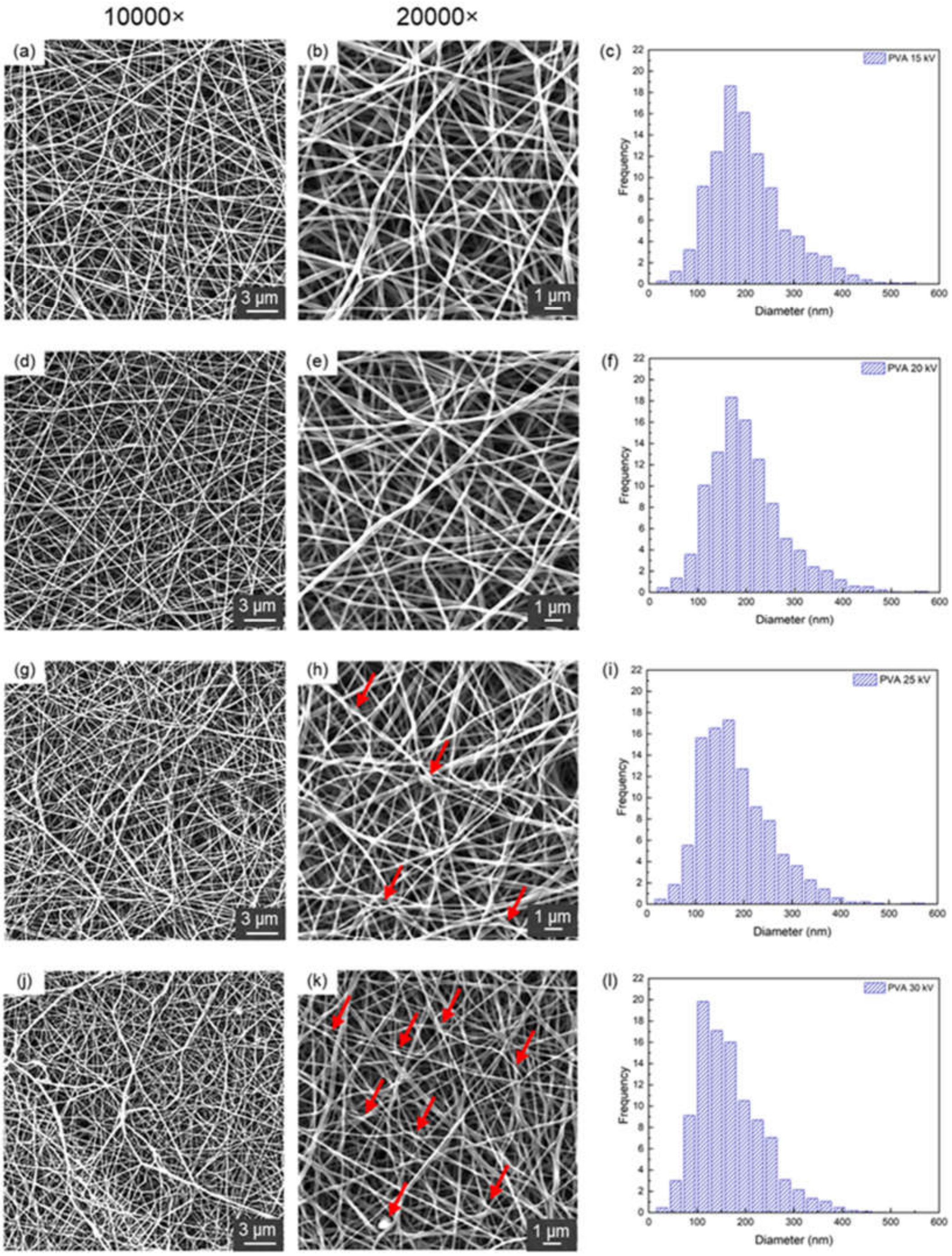

Figure S1. SEM images of PVA nanofibers electrospun from 10 wt% polymer solutions at applied voltages of (a, b) 15 kV, (d, e) 20 kV, (g, h) 25 kV, and (j, k) 30 kV. Images acquired at magnifications of 10,000× (a, d, g, j) and 20,000× (b, e, h, k). The corresponding fiber diameter distributions are shown in panels (c, f, i, l). Increasing voltage reduced mean fiber diameter, while bead defects became evident at 25–30 kV (red arrows).

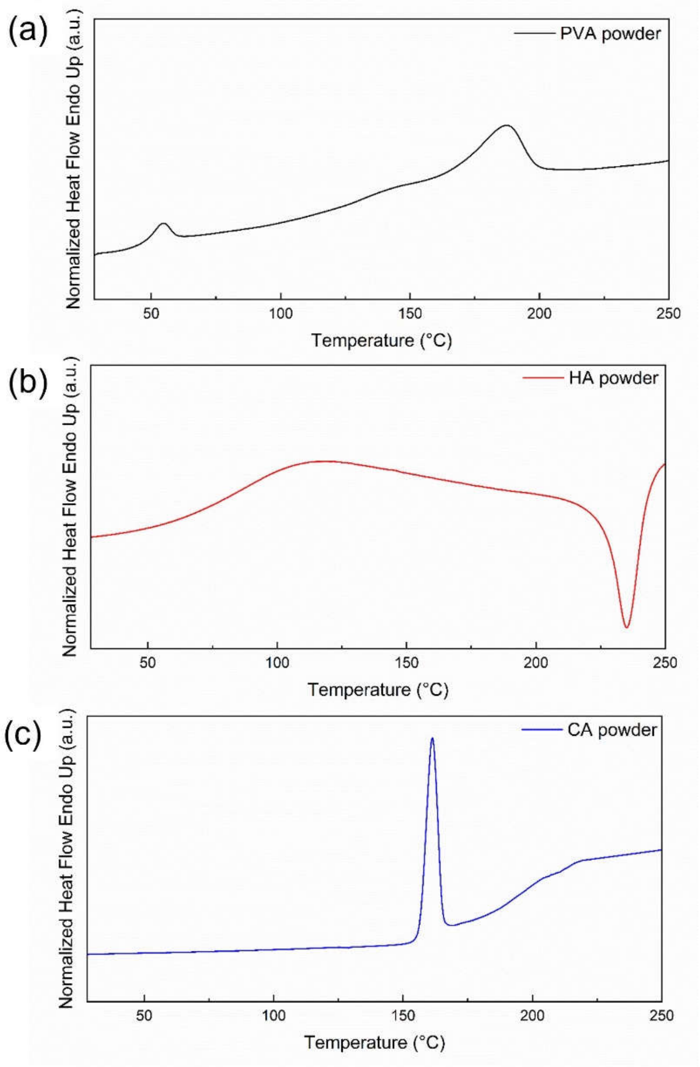

Figure S2. DSC thermograms of (a) PVA, (b) HA, and (c) CA as supplied by the manufacturer (powder form) at a heating rate of 10 °C/min.
